# Hypothalamic inflammation is reversed by endurance training in anorectic-cachectic rats

**DOI:** 10.1186/1743-7075-8-60

**Published:** 2011-08-24

**Authors:** Fábio S Lira, Alex S Yamashita, Jose C Rosa, Fábio L Tavares, Erico Caperuto, Luiz C Carnevali, Gustavo D Pimentel, Ronaldo VT Santos, Miguel L Batista, Alessandro Laviano, Filippo Rossi-Fanelli, Marília Seelaender

**Affiliations:** 1 Cancer Metabolism Research Group, Institute of Biomedical Sciences, University of São Paulo (USP), São Paulo, SP - Brazil; 2 Department of Physiology, Division of Nutrition Physiology, Federal University of São Paulo (UNIFESP), São Paulo, SP - Brazil; 3 Department of Bioscience, Federal University of São Paulo, Baixada Santista, Campus, São Paulo, Brazil; 4 Department of Clinical Medicine, Sapienza University of Rome, Rome - Italy

**Keywords:** anorexia, cancer cachexia, hypothalamus, exercise training, cytokines, inflammation

## Abstract

**Aim:**

We tested the effects of a cancer cachexia-anorexia sydrome upon the balance of anti and pro-inflammatory cytokines in the hypothalamus of sedentary or trained tumour-bearing (Walker-256 carcinosarcoma) rats.

**Methods:**

Animals were randomly assigned to a sedentary control (SC), sedentary tumour-bearing (ST), and sedentary pair-fed (SPF) groups or, exercised control (EC), exercised tumour-bearing (ET) and exercised pair-fed (EPF) groups. Trained rats ran on a treadmill (60%VO_2max_) for 60 min/d, 5 days/wk, for 8 wks. We evaluated food intake, leptin and cytokine (TNF-α, IL1β) levels in the hypothalamus.

**Results:**

The cumulative food intake and serum leptin concentration were reduced in ST compared to SC. Leptin gene expression in the retroperitoneal adipose tissue (RPAT) was increased in SPF in comparison with SC and ST, and in the mesenteric adipose tissue (MEAT) the same parameter was decreased in ST in relation to SC. Leptin levels in RPAT and MEAT were decreased in ST, when compared with SC. Exercise training was also able to reduce tumour weight when compared to ST group. In the hypothalamus, IL-1β and IL-10 gene expression was higher in ST than in SC and SPF. Cytokine concentration in hypothalamus was higher in ST (TNF-α and IL-1β, p < 0.05), compared with SC and SPF. These pro-inflammatory cytokines concentrations were restored to control values (p < 0.05), when the animals were submitted to endurance training.

**Conclusion:**

Cancer-induced anorexia leads towards a pro-inflammatory state in the hypothalamus, which is prevented by endurance training which induces an anti-inflammatory state, with concomitant decrease of tumour weight.

## Introduction

Anorexia-cachexia and its consequences are detrimental and considered to be the direct cause of up to 20% of cancer deaths [1]. Adipose tissue atrophy is a hallmark of cancer cachexia, up to an 85% decrease in body fat being reported in lung cancer patients, leading to hyperlipidaemia and insulin resistance as well as complicating anti-tumour therapies. Loss of fat stores cannot be explained by reduced appetite alone as it often precedes the onset of anorexia and is more severe in an animal model of cachexia than during food restriction [2,3]. Evidence has accumulated that the disease progress triggers catabolic responses that override anabolism in the peripheral tissues [4].

The role of pro-inflammatory cytokines, and particularly of interleukin-1 (IL-1β) and tumour necrosis factor alpha (TNF-α) in the pathogenesis of the anorexia-cachexia syndrome has been recognized for many years [5]. In tumour-bearing rats with anorexia, hypothalamic IL-1 mRNA expression is significantly increased [6] and augment of circulating levels of many pro-inflammatory cytokines [7], which are able to permeate the blood-brain-barrier [8]. On the other hand, anti-inflammatory cytokines, in special IL-10 [9,10], modulate the outcome of inflammatory diseases.

Recent evidence sheds light on how cytokines produced by the tumour or by the peripheral tissues reach the brain and modulate appetite. Likewise, several studies have shown that the increase of hypothalamic pro-inflammatory cytokine content is the main factor responsible for the potent stimulation of the catabolic neuropeptide, proopiomelanocortin (POMC), and diminished secretion of the anabolic neuropeptide Y (NPY) [5,6].

It was our aim to examine hypothalamic cytokine profile in the Walker 256 model and to evaluate the capacity of chronic moderate exercise to interfere in this aspect, bearing in mind its anti-inflammatory potential.

## Materials and Methods

### Animals

A total of 39 male 6 weeks-old *Wistar *rats (weighing ~250 g), obtained from the Animal Breeding Unit of the Institute of Biomedical Sciences, University of São Paulo, were used. They were housed, five per cage, receiving food and water *ad libitum*, in an animal room under 12 h light-dark cycle, at 22°C ± 1°C and 60 ± 5% humidity. The experiments were carried out after acclimation for a week, and in accordance with the Guide for the Care and Use of Laboratory Animals, published by the US National Institute of Health (NIH Publication No. 85-23, revised 1996). The protocol was approved by the Committee of Ethics in Animal Experimentation of the Institute of Biomedical Sciences, University of São Paulo (041/2005).

### Experimental design

The rats were randomly assigned to either a sedentary or an exercised group, and then subdivided into the following groups: sedentary control (SC, n = 7), sedentary tumour-bearing (ST, n = 7), sedentary pair-fed (SPF, n = 7), or: exercised control (EC, n = 6), exercised tumour-bearing (ET, n = 6), and exercised pair-fed (EPF, n = 6). The trained groups ran for 2 weeks in order to adapt to the treadmill. After that period, they were submitted to the 8-weeks training protocol (described below). At the end of the 6th week of training, Walker 256 tumour cells were inoculated in ST and ET. After the fourteenth day, all the animals were killed by decapitation, without anesthesia.

### Training protocol

The rats were submitted, as described by Lira *et al *[11], to a pre-training period of 2 weeks, during which they ran progressively from 15 to 60 min at 10 m/min. During the following training period of 8 weeks (training 5 days/weeks), animals exercised on a motorised treadmill (Enlaup, São Paulo, Brazil), at 24°C and 80% humidity. Running velocity was increased to 20 m/min in the last 2 weeks, and intensity maintained between 60 and 65% VO2max, as determined periodically in an Oxymax Columbus System (Columbus Instruments, Columbus, OH). The sensor response time was 4 s at 80 mL/min flow; the repeatability was ± 0.01% O2 at constant temperature and pressure, and the drifty was lower than 0.01% O2/h. Due to the ready adaptation of to the animals treadmill, and also to the fact that exercise sessions were performed during the period of activity of the animals, no reinforcement was required. After a resting period of 24 hours after the last workout session, animals were sacrificed.

### VO_2max _determination

VO2 max was determined by having each rat perform a maximal exercise test adapted from Lira *et al *[11]. The parameters were measured using the Oxymax gas analysing system for small animals (Columbus Instruments). The test was always carried out after a 1-day recovery period. A baseline measurement was taken before the beginning of the training protocol. The volume of the supplied air was 4.5 L/min. The gas analysis was calibrated with a reference gas mixture before each test. The VO2 max test protocol involved stepwise increasing of the treadmill speed as follows: after a 15-min period of acclimation, the treadmill was started at 10 m/min, and the speed was incrementally increased 5 m/min every 3 min until the rat reached exhaustion. Exhaustion was defined as spending time on the shocker plate without attempting to reengage the treadmill within 15 s. The highest VO_2max _measured at each workload was taken as a measure of each rat's running economy (VO_2submax_) for that workload, and at the last step, as VO_2max_.

### Tumour cell inoculation

Walker-256 carcinosarcoma (2 × 10^7 ^cells/rat) cells in 1 mL PBS solution were injected subcutaneously, into the right flank of the rats, according to Seelaender *et al *[12]. All experiments were carried out on the fourteenth day following tumour cell injection. After sacrifice, the tumour was dissected and weighed.

### Evaluation and food intake

Food intake of the six groups was assessed every morning during the whole experimental period.

### Hormone concentration

Blood was collected and serum samples were separated after allowing blood to clot on ice. Serum was stored frozen at -80°C for analysis. Retroperitoneal (RPAT) and Mesenteric (MEAT) adipose tissue, and serum leptin was quantified using a RIA commercial kit (Genese^®^, Brazil). Results are expressed as ng/mL (plasma) and ng/mg (tissues).

### Analysis of gene expression

Total RNA were obtained from aliquots of 100 mg of adipose tissue (RPAT and MEAT) and of the hypothalamus of the rats by TRIZOL^® ^reagent extraction, as previously described [13]. RNA concentration was determined spectrophotometrically (Beckman DU 640, Fullerton, Calif., USA). A 33-μl assay mix containing 3 μg RNA, 10 U placental RNAse inhibitor, 2 μl oligo(dt), 2 μl dNTP (10 nmol), 2 μl dithiothreitole, 10 U Moloney-murine leukaemia virus reverse transcriptase (Invitrogen, USA), and 4 μl 10× reaction buffer (100 mM TRIS-HCl pH 8.3, 500 mM KCl, 150 mM MgCl2 in nuclease-free water) was used to produce cDNA. The RT-mixture was incubated at 80°C for 3 min, followed by 21°C for 10 min, 42°C for 30 min and then 99°C for 10 min. The obtained product (2 μl) was fractionated in 1% agarose and ethidium bromide gel to assess the quality of the reaction. The primers were designed with regard to the published Genebank sequences.

### Real time PCR

Primers sets for rat proteins above were designed using Primer Express software v2.0 (Applied Biosystems, Foster City, California). The results for mRNA (TNF-α, IL-1β and IL-10 in the hypothalamus, and leptin RPAT and MEAT) concentrations are expressed as ratio over GAPDH (glyceraldehyde 3-phosphate dehydrogenase), which was amplified as housekeeping gene. For each sample, PCR was performed in duplicate in a 25-μl reaction volume of 5-20 ng of cDNA, 12.5 μl Syber Green Master Mix (Applied Biosystems), and 200 nM of each primer. PCR analyses were carried out using the following cycle parameters: 50°C for 2 min, 95°C for 10 min, followed by 40 cycles of 95°C for 15 s, and 60°C for 1 min. Fluorescence was quantified and analysis of amplification plots was performed with the AB 7300 Sequence Detector System (Applied Biosystems). Results are expressed using the comparative cycle threshold (Ct) method as described by the manufacturer.

### Immunoassays

Hypothalamus samples were carefully rinsed in ice-cold 0.9% NaCl to remove any blood contaminants, snap-frozen in liquid nitrogen and stored at -80°C. Frozen tissue (0.1-0.3 g) was homogenized in RIPA buffer (0.625% Nonidet P-40, 0.625% sodium deoxycholate, 6.25 mM sodium phosphate, and 1 mM ethylene-diamine tetraacetic acid at pH 7.4) containing 10 μg/ml of a protease inhibitor cocktail (Sigma-Aldrich, St. Louis, Missouri). Homogenates were centrifuged at 12.000 *g *for 10 min at 4°C, the supernatant was saved, and protein concentration was determined as described by Bradford *et al *[14] (Bio-Rad, Hercules, California) with bovine serum albumin as a reference. Quantitative assessment of TNF-α, IL-1β and IL-10 proteins was carried out with ELISA (DuoSet ELISA, R & D Systems, Minneapolis, MN). For TNF-α (DY510), IL-1β (DY501) and IL-10 (DY522) assay sensitivity was found to be 5.0 pg/mL in the range of 31.2-2000 pg/mL. The intra- and inter-assay variability of the TNF-α and IL-1β kits were 2.7-5.2, and 4.9-9.5%, respectively. Assay sensitivity for IL-10 was 10 pg/mL in the range from 31.2-2000 pg/mL. The intra-assay variability of the IL-10 kit was 2.0-4.2%, and its inter-assay variability was of 3.3-6.4%. All samples were run as duplicates and the mean value was reported.

### Statistical analysis

The statistical analysis was performed using the GraphPad Prism statistics software package version 5.0 for Windows (GraphPad Software, San Diego, CA, USA). Data are expressed as means ± SEM. Implementation of the Kolmogorov-Smirnov test revealed that the results of experiments were distributed normally. Post-training measurements were analysed by 2-way ANOVA of 2 × 2 design, whose data were partitioned into main effects (sedentary vs. exercise group effects, A; and tumour-bearing vs. control group effects, B). The interaction effects consisted of A × B. When a significant *F *value was found by 2-way ANOVA, a Tukey post hoc test was performed to demonstrate all pairwise multiple comparisons between the means. The 0.05 probability level was considered to indicate statistical significance.

## Results

### Food intake

Table 1 shows the values of cumulative food intake which were decreased in the cachectic animals (26%, p < 0.05), when compared with control animals. Exercise training induced a decrease in food intake in EC (training effect) (20%, p < 0.05), when compared with SC (Table 1).

**Table 1:** Food intake during 14 days of tumour and leptin levels after 8 weeks of training

	Food intake (pre-tumour)	Food intake (1° wk tumour)	Food intake (2° wk tumour)	Cumulative food intake (14 dys tumour)	Leptin (ng/mL)
**SC**	23.68 ± 2.23	23.01 ± 1.80	22.52 ± 1.87	314.8 ± 24.2	0.58 ± 0.13
**ST**	22.65 ± 0.68	18.55 ± 0.57*	17.76 ± 0.80*	254.1 ± 8.8*	0.29 ± 0.06*
**SPF**					0.21 ± 0.02*
**EC**	20.79 ± 1.44	19.10 ± 0.98*	15.35 ± 0.70*	241.1 ± 9.3*	0.57 ± 0.09
**ET**	19.30 ± 1.95	18.99 ± 1.06	16.93 ± 1.35	257.1 ± 15.5	0.37 ± 0.06
**EPF**					0.16 ± 0.009#

Results are expressed as mean value ± SEM. SC: sedentary control (n = 7), ST: sedentary tumour-bearing (n = 7), SPF: sedentary pair-fed (n = 7), EC: trained control (n = 6), ET: trained tumour-bearing (n = 6), EPF: trained pair-fed (n = 6). *p < 0.01 vs. sedentary control; #p < 0.001 vs. sedentary pair-fed.

### Leptin serum levels

Leptin serum concentrations were decreased in ST (45%, p < 0.05) and SPF (61%, p < 0.05), when compared with SC. There was no evidence of an effect on leptin levels induced by exercise training (Table 1).

### Leptin expression and content in RPAT and MEAT

Table 2 shows leptin gene and protein expression in the RPAT and MEAT pads. Leptin gene expression in RPAT was increased in SPF compared with SC and ST (p < 0.001), and decreased in MEAT of ST, in relation SC (p < 0.001). Leptin levels as evaluated by ELISA were reduced in RPAT and MEAT of ST, when compared with SC (p < 0.001). These parameters were restored to control values (p < 0.05) when the animals were submitted to the exercise training protocol.

**Table 2:** Leptin mRNA and protein expression in the RPAT and MEAT rats

RPAT	MEAT	
Gene expression (U.A)		

**SC**	**0.76 ± 0.17**	**1.03 ± 0.13**

**ST**	**0.74 ± 0.18**	0.65 ± 0.05*

**SPF**	4.40 ± 1.80*#@	**0.80 ± 0.05**

EC	0.46 ± 0.12	0.64 ± 0.11

ET	0.76 ± 0.04	0.36 ± 0.06

EPF	1.06 ± 0.20	0.52 ± 0.15

**Protein expression (ng.mg tissue)**		

**SC**	**1.20 ± 0.17**	**0.202 ± 0.02**

**ST**	0.63 ± 0.067*	0.042 ± 0.02*

**SPF**	0.63 ± 0.08*	0.044 ± 0.01*@

EC	1.45 ± 0.15@	0.250 ± 0.05

ET	1.18 ± 0.36@#	0.141 ± 0.009#

EPF	0.95 ± 0.040	0.174 ± 0.03

Results are expressed as mean value ± SEM. SC: sedentary control (n = 7), ST: sedentary tumour-bearing (n = 7), SPF: sedentary pair-fed (n = 7), EC: trained control (n = 6), ET: trained tumour-bearing (n = 6), EPF: trained pair-fed (n = 6). *p < 0.01 vs. sedentary control; #p < 0.001 vs. sedentary pair-fed. *p < 0.001 significantly different from control values.

# p < 0.001 significantly different from sedentary tumour values.

@ p < 0.001 significantly different from exercise pair-fed values.

### IL-1β, TNF-α and IL-10 expression and content in the hypothalamus

**Figure 1:**
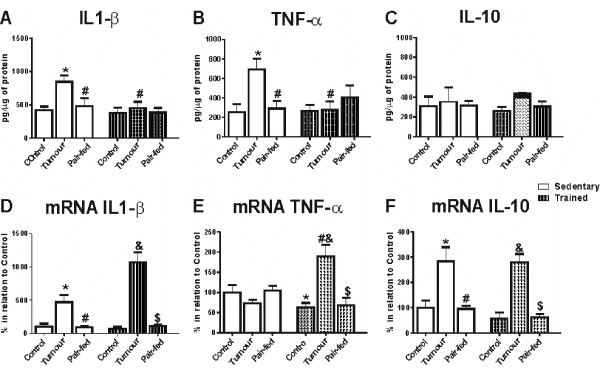
**Hypothalamic cytokine levels and it respective gene expression interleukin 1β (IL-1β), tumour necrose factor alpha (TNF-α), and interleukin 10 (IL-10) in rats. **Results are expressed as mean value ± SEM. SC: sedentary control (n = 7), ST: sedentary tumour-bearing (n = 7), SPF: sedentary pair-fed (n = 7), EC: trained control (n = 6), ET: trained tumour-bearing (n = 6), EPF: trained pair-fed (n = 6). *p < 0.01 vs. sedentary control; #p < 0.01 vs. sedentary tumour-bearing; &p < 0.01 vs. trained control; $p < 0.01 vs. trained tumour-bearing**.**

Cytokine protein levels were increased in the cachectic animals (IL-1β 1.1 fold, p < 0.05; TNF-α 1.8 fold, p < 0.01 and IL-10 1.3 fold, p < 0.01) when compared with control and pair-fed animals (Figure 1A, B and 1C). Endurance training decreased these values in ET (training effect) in relation to ST (TNF-α -60%, p < 0.05 and IL-1β -35%) (Figure 1A and 1B). IL-10 levels were not modulated by endurance training (Figure 1C).

Cachexia modified the hypothalamic gene expression of IL-1β and IL-10, which were increased in ST and ET, when compared with SC, SPF, EC and EPF, respectively (p < 0.01) (Figure 1D and 1F). TNF-α mRNA was increased in ET in relation to ST, and in EPF and EC (p < 0.01) (Figure 1E). In addition, TNF-α mRNA was decreased in EC, when compared with SC (p < 0.01) (Figure 1E).

**Figure 2:**
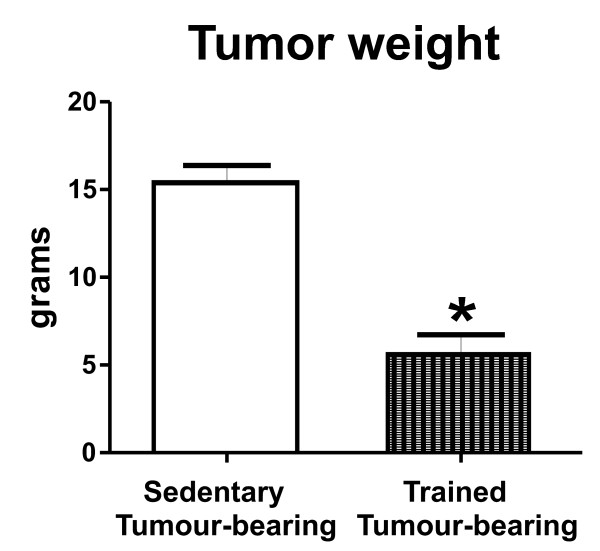
**The effect of exercise training on tumour weight in cancer-induced anorexia rats**. Results are expressed as mean value ± SEM.ST: sedentary tumour-bearing (n = 7) and ET: trained tumour-bearing (n = 6). *p < 0.01 vs. sedentary tumour-bearing.

Furthermore, endurance exercise was efficient in reducing total tumour weight (3-fold, p < 0.01) in ET animals, when compared with their sedentary counterparts (15.4 g sedentary vs. 5.5 g exercise) (Figure 2).

## Discussion

We presently demonstrate a higher protein content for TNF-α and IL-1β in the hypothalamus of rats with anorexia-cachexia associated with cancer. These pro-inflammatory cytokines are known to modulate the expression and secretion of neuropeptides involved in the control of feeding behavior [5,6,15-18]. These results are in agreement with our hypothesis that Walker 256- bearing rats present hypothalamic inflammation.

Food intake regulation under physiological situations is modulated among others aspects, by visceral adiposity, through the secretion of leptin by the white adipose tissue [19]. This hormone plays a direct action in the central nervous system, where it binds to OB-Rb receptor sending signals to the hypothalamic arcuate nucleus, inducing increased anorexigenic neuropeptide expression (POMC and cocaine- and amphetamine-regulated transcript-CART) and decreasing food intake. In the arcuate nuclei the orexigenic neuropeptide NPY plays a pivotal role in the control of food intake, as its expression is regulated especially by leptin; higher concentrations of leptin inhibit NPY action, reducing food intake and, when the opposite occurs (eg. leptin decreases), NPY expression is higher, stimulating food intake [5,6,15-18]. This balance is lost during cachexia, as even in front of a markedly reduced concentration of leptin in the plasma in tumour-bearing animals [20], anorexia is still present.

Previous studies from our laboratory have shown that animals injected with the Walker-256 tumour are anorectic, show reduced plasma leptin concentration, as well as reduced concentration of leptin in different depots of adipose tissue [20]. These alterations are accompanied by the presence of a mononuclear infiltrate in the fat pads, suggesting an important role of these cells in the induction of alterations of leptin levels. Therefore, we may conclude that in the Walker 256 model food intake decrease is probably associated with one such reduction of leptin levels concomitant to increased local inflammatory cytokine expression.

Chronic exercise presents itself as a possible low-risk therapy for cancer patients, as it has been show in animal models to prevent or even reverse cachexia, and therefore potentially improve patients life quality [10,11,21-23]. Previous studies by our group have shown that the anti-inflammatory effect of exercise seems to be, as expected, more evident under the presence of non-physiological conditions, such as cancer cachexia [11,24] and malnourishment [25]. The mechanisms by which regular exercise offers its protection are through the regulation of body weight and reduction of inflammation in the plasma and in the adipose tissue [26-28]. However, the response depends on the intensity, frequency and volume of the exercise, yet several studies demonstrate that moderate exercise may also modulate immune response [29-32]. Recently, Hanahan & Weinberg [8] have provided evidence that leptin and pro-inflammatory cytokines are modulated by exercise.

Chennaoui et al. [33] showed an effect of exercise training in the concentrations of IL-1β, IL-6 and IL-1ra in the central nervous system of rats, with reduction of these pro-inflammatory cytokines, suggesting an important role of exercise in the control of inflammation in the brain. Interestingly, a single session of exercise was show to induce the expression of IL-6 in the nervous tissue [34]. Thus, it important to make clear that exercise may modulate the production of brain cytokines and affect appetite controlling systems at both short- and long-term. Several studies also show that after an acute session of exercise there is an exponential increase in IL-6 levels (over 100-fold), which likewise, depends on the variables of exercise, such as intensity, duration, muscle recruiting and individual aerobic capacity [35,36]. This modification is followed by increased IL1-ra and IL-10 levels, which may be induced by IL-6 [26].

There are enhanced IL1-ra and IL-10 levels after a single bout of exercise, contributing to anti-inflammatory *milieu*, yet to our knowledge few studies have evaluated the behaviour of IL-1ra and IL-10 after chronic exercise. Although hypothalamic IL-10 levels were not modified.

Additionally, eight weeks of endurance training decreased tumour weight in trained rats (mean. 5.5 g). when compared to sedentary rats (mean. 15.4 g). The mechanism by which endurance training inhibits tumour growth yet is unknown. However, is clear that increase of immune system function by moderate exercise is able to activate antitumoural activity [37], thereby reducing tumour weight.

In summary, our results shown that in the hypothalamus of sedentary tumour-bearing animals increase in TNF-α and IL-1β levels exacerbated the disturbances that affect the reduction of food intake and worsened the framework of cancer-induced anorexia. Moreover, the endurance training program restored the basal concentrations of pro-inflammatory cytokines and reduced tumour weight.

## Conflicts of interests

The authors declare that they have no competing interests.

## Authors' contributions

FSL, ASY, JCR, FLT, EC, LCC, GDP, RVTS, MLB, AL, FRF and MS participated of sample collected, assay samples, design of the study and performed the statistical analysis, writing and discussion of paper. All authors read and approved the final manuscript.
